# *OsMYBR1*, a 1R-MYB Family Transcription Factor Regulates Starch Biosynthesis in Rice Endosperm

**DOI:** 10.3390/life15060962

**Published:** 2025-06-16

**Authors:** Kunyong Huang, Long Chen, Guiai Jiao, Zheyan Ruan, Xinwei Li, Shaoqing Tang, Peisong Hu, Xiangjin Wei

**Affiliations:** 1 State Key Laboratory of Rice Biology and Breeding, China National Center for Rice Improvement, China National Rice Research Institute, Hangzhou 310006, China; huangkunyonk@163.com (K.H.); chenlong@caas.cn (L.C.); jiaoguiai@caas.cn (G.J.); skyhesea@163.com (Z.R.); lixinwei162013@163.com (X.L.); tangshaoqing@caas.cn (S.T.); 2 Environment-Friendly Crop Germplasm Innovational and Genetic Improvement Key Laboratory of Sichuan Province, Chengdu 610066, China

**Keywords:** rice, starch synthesis, amylose content, *OsMYBR1*, rice quality

## Abstract

Starch is the primary component of the endosperm and plays a crucial role in rice quality. Although the enzymes involved in starch synthesis have been extensively studied, the transcription factors that regulate these enzymes remain largely unknown. Here, we identified a MYB family transcription factor, *OsMYBR1*, that regulates starch biosynthesis in rice. *OsMYBR1* is highly expressed during endosperm development. Mutations of *OsMYBR1* result in reduced grain thickness and a decrease in 1000-grain weight. The endosperm of *osmybr1* mutants exhibit rounded and loosely packed starch granules, decreased amylose content, altered fine structure of amylopectin, and modified physicochemical properties. The analysis of RT-qPCR showed that the expression of several starch-synthesis enzyme-coding genes (SSEGs), including *OsGBSSⅠ*, *OsAGPL1*, *OsAGPL2*, *OsBEⅡb*, *OsISA1*, *PHOL*, and *OsSSⅢa*, is altered in *osmybr1* mutants. Further experiments indicated that OsMYBR1 directly binds to the promoters of *OsGBSSⅠ*, *OsAGPL1*, *OsAGPL2*, *OsISA1*, *OsBEⅡb*, and *PHOL*, resulting in an increase in the expression of *OsGBSSⅠ* but a decrease in the expression of *OsAGPL2*, *OsISA1*, and *OsSSⅢa*. In contrast, *OsMYBR1*-overexpressing endosperm appears normal, with starch granule morphology, increased amylopectin content, and improved alkali spreading value, indicating enhanced rice eating and cooking quality (ECQ). These findings suggest that the overexpression of *OsMYBR1* could be a promising strategy for improving rice ECQ.

## 1. Introduction

Rice (*Oryza sativa* L.) is a vital staple food worldwide, with starch comprising more than 80% of the rice endosperm [1]. Starch is composed of linear amylose and highly branched amylopectin, and the ratio of these components, along with their hierarchical physicochemical properties, determines the rice quality [2,3]. Key indicators for evaluating and estimating rice eating and cooking quality (ECQ) include amylose content (AC), gel consistency (GC), and gelatinization temperature (GT).

Starch biosynthesis is a complex biochemical process involving multiple coordinated enzymes [4,5]. Amylose synthesis is primarily controlled by granule-bound starch synthase Ⅰ (GBSSⅠ), encoded by the *Waxy* gene. Generally, loss-of-function of OsGBSSⅠ generated glutinous rice [6,7]. Furthermore, genome editing the *Waxy* promoter or 5′-UTR-intron has been shown to fine-tune amylose levels and improved rice quality [8]. Recently, novel *Wx* alleles generated through base editing have been developed to enhance rice grain quality [9]. In contrast, amylopectin synthesis requires a series of enzymes, including soluble starch synthases (SSs), starch branching enzymes (SBEs), and debranching enzymes (DBEs). SSs are responsible for the elongating of amylopectin molecules, SBEs create α-1,6-glycoside branch points, and DBEs remove improper branch chains by hydrolyzing these bonds. Reduced expression or loss-of-function of enzymes, such as OsSSⅠ, OsSSⅡa, OsSSⅢa, OsBEⅡb, and OsISA1, disrupts starch biosynthesis and granule formation, resulting in abnormal endosperm development [10,11,12,13,14,15,16,17].

The enzymes involved in starch synthesis are regulated by various transcription factors. The MYC transcription factor OsBP-5 forms a heterodimer with the ethylene-responsive element binding protein OsEBP-89, enhancing *Waxy* gene transcription [18]. The nuclear factor Y family gene NF-YB1 forms a heterotrimer complex with NF-YC12 and bHLH144, directly activating the *Waxy* gene to regulate amylose content and grain quality [19]. Additionally, the NAC transcription factors OsNAC20 and OsNAC26 directly transactivate the expression of genes involved in starch and storage protein synthesis, including OsSSⅠ, OsPUL, DPE1, GluA1, GluB4/5, α-globulin, and 16 kD prolamin [1]. OsNAC24 acts as a transcriptional activator targeting the promoters of six starch-synthesis enzyme-coding genes (SSEGs): *OsGBSSⅠ*, *OsSBEⅠ*, *OsAGPS2*, *OsSSⅠ*, *OsSSⅢa*, and *OsSSIVb* [5]. Among bZIP family transcription factors, OsbZIP58 binds promoters of six starch synthesis genes (*OsAGPL3*, *Wx*, *OsSSⅡa*, *SBE1*, *OsBEⅡb*, and *ISA2*) to regulate their expression [20]. OsbZIP60 also activates the expression of several starch synthesis-related genes (*GBSSI*, *AGPL2*, *SBEI*, and *ISA2*) and storage protein synthesis-related genes (*OsGluA2*, *Prol14*, and *Glb1*) [21]. Conversely, the AP2/EREBP family transcription factor *RSR1* negatively regulates the expression of several SSEGs, influencing amylose content and amylopectin structure [22].

MYB transcription factors constitute one of the largest plant-specific families, playing key roles in growth, development, metabolism, hormone signaling, disease resistance, and stress tolerance [23,24,25,26,27]. To date, more than 155 MYB genes have been identified in rice [23]. In maize, overexpression of *ZmMYB115* significantly suppressed the expression of *Du1*, a key enzyme involved in the elongation of amylopectin chains, while enhancing *Wx* promoter activity related to amylose synthesis in the maize endosperm [28]. *ZmMYB14* binds to the promoter of *ZmBT1*, a transporter of adenosine diphosphate-glucose, activating its expression and regulating maize starch synthesis [29]. However, despite these findings, there are few reports on the role of MYB family transcription factors in rice starch synthesis.

In this study, we identified 137 MYB family proteins in rice using HMMER searches. Expression analysis revealed that nine MYB genes are highly expressed in seeds. Further analysis identified the 1R-MYB transcription factor *OsMYBR1* (LOC_Os10g20990), which is highly expressed in the immature rice endosperm. We hypothesize that *OsMYBR1* plays a crucial regulatory role in starch biosynthesis during endosperm development. Consistent with this, loss-of-function mutants in *OsMYBR1* altered the expression of several SSEGs, moderately impacting starch biosynthesis, composition, structure, starch granules, and physicochemical properties. Conversely, overexpression of *OsMYBR1* improved the amylopectin content without affecting endosperm appearance or starch granule morphology, ultimately enhancing rice quality.

## 2. Materials and Methods

### 2.1. Plant Growth Conditions and Phenotypic Characterizations

The *Oryza sativa* L. ssp. *japonica* rice cultivar ‘Nipponbare’ (NIP) was used for transformation experiments. Wild-type NIP and all the transgenic plants were grown at the experimental fields in Hangzhou, Zhejiang Province, China, during the summer season. The seed length, seed width, seed thickness, 1000-grains-weight, chalky grain rate, and chalkiness degree of NIP, mutants, and overexpression lines were examined using a seed phenotyping system (Wanshen, Hangzhou, China).

### 2.2. Bioinformatics and Phylogenetic Analysis of OsMYBR1 Protein

The conserved domains of OsMYBR1 were predicted in NCBI (https://www.ncbi.nlm.nih.gov/Structure/cdd/wrpsb.cgi (accessed on 5 April 2025)). Orthologues of OsMYBR1 were identified via the Gramene database (http://www.gramene.org/ (accessed on 5 March 2025)) and protein sequences downloaded from NCBI’s non-redundant protein database using BLASTp (https://blast.ncbi.nlm.nih.gov/Blast.cgi (accessed on 5 March 2025)) with OsMYBR1 as the query. A total of 137 MYB family proteins were identified (listed in Supplemental Table S1).

Phylogenetic analysis was performed on full-length OsMYBR1 and its homologous proteins in *Zea mays*, *Triticum urartu*, *Triticum aestivum*, *Glycine max*, *Arabidopsis thaliana*, rice (*Oryza sativa*), barley (*Hordeum vulgare*), *Setaria viridis*, *Paspalum notatum*, and *Digitaria exilis*. Sequences were aligned using MUSCLE in the MEGA 7.0 software [30]. The phylogenetic tree was constructed using the neighbor-joining method with 1000 bootstrap replicates.

### 2.3. Gene Expression Analysis of OsMYBR1

Transcript abundance of *OsMYBR1* in root, stem, leaf, anther, pistil, panicle, and seed was acquired from RiceData (https://www.ricedata.cn/gene/ (accessed on 5 March 2025)). The qRT-PCR was used to analyze the expression pattern of *OsMYBR1*. The tissues of root, stem, leaf, spikelet, and developmental seeds (3, 10, 18, and 24 days after flowering (DAF)) were collected form NIP. Here, 3 DAF represented the early developmental stage, 10 DAF and 18 DAF corresponded to storage product accumulation stage, and 24 DAF indicated the maturation stage. Total RNA was extracted using the TRIzol reagent (Carlsbad, CA, USA) and reverse transcribed into cDNA with ReverTra Ace qPCR RT Kit (Toyobo, Osaka, Japan). SYBR Green Real-Time PCR Master Mix (Toyobo) was used for qRT-PCR analysis. The rice Ubiquitin gene (Os03g0234200) served as the internal control. Relative expression was calculated using the comparative CT method [21]. All primer sequences used in this analysis are listed in Supplemental Table S2.

### 2.4. Subcellular Localization of OsMYBR1 in Rice Protoplasts

The coding sequence (CDS) of *OsMYBR1* was fused to the N-terminus of enhanced green fluorescent protein (eGFP) under the control of the 35S promoter, generating the *p35S:OsMYBR1-eGFP* construct. Free eGFP was used as a control. Both constructs were transiently expressed in rice protoplasts. After 16 h incubation, fluorescence was observed using a Zeiss LSM980 (Jena, Germany). DAPI staining served as a nuclear marker. Primer sequences are in Supplemental Table S2.

### 2.5. Construction of Gene Overexpression and Gene Knockout in Rice

The CDS of OsMYBR1 was cloned from NIP into the pCAMBIA1305 binary vector for overexpression. For knockout, the CRISPR/Cas9 system was used, with guide RNA editing sequences inserted to the BGK03 vector (Biogle, Changzhou, China). Primers and sequencing details are provided in Supplemental Table S2.

### 2.6. Scanning Electron Microscopy

Mature rice seeds were dried completely at 37 °C and cut longitudinally. Transverse sections were fixed, gold-coated, and observed under scanning electron microscopy (SEM) to examine starch grain morphology.

### 2.7. Physicochemical Properties of Endosperm Starch

The total starch and amylose content of polished seeds were measured using Megazyme assay kits K-TSTA and K-AMYL (Megazyme, Wicklow, Ireland). Total protein content was determined following Kang et al. [31]. The Alkali spreading value (ASV) assay evaluated the degree of spreading of milled rice in a 1.7% KOH solution after 23 h according to Cruz et al. [32]. ASV scores are generally categorized into four groups: (1) high GT = ASV score 1–2, (2) high intermediate GT = ASV score 3, (3) intermediate GT = ASV score 4–5, and (4) low GT = ASV score 6–7 [33].

Gelatinization temperature was measured by differential scanning calorimetry (DSC) (Mettler-Toledo, Columbus, OH, USA). We measured 5 mg of rice flour in the pan, added 10 μL ddH_2_O, and the pan was sealed. Then the samples were heated from 25 to 120 °C at 10 °C/min. The thermodynamic characteristics of the rice flour, including onset temperature, peak temperature, conclusion temperature, and enthalpy (ΔH), were recorded.

Amylopectin chain length was determined via isoamylase digestion (Sigma-Aldrich, St. Louis, MO, USA) followed by capillary electrophoresis.

### 2.8. Yeast 1-Hybrid (Y1H), Dual-Luciferase Reporter and Electrophoresis Mobility Shift (EMSA) Assay

For Y1H assay, the CDS of OsMYBR1 was fused to the GAL4 AD structure domain in the pB42AD vector, and the promoter sequence of *OsGBSSⅠ*, *OsAGPL1*, *OsAGPL2*, *OsPHOL*, *OsBEⅡb*, *OsISA1*, and *OsSSⅢa* was cloned into pLacZi vectors. Vectors were transformed into yeast strain EGY48 and cultured on SD/-Ura-His medium containing X-Gal. For dual-luciferase reporter assay (LUC), the promoter of *OsGBSSⅠ*, *OsAGPL1*, *OsAGPL2*, *OsPHOL*, *OsBEⅡb*, *OsISA1*, and *OsSSⅢa*, was cloned into the pGreenⅡ 0800-LUC vector to create reporter constructs and the CDS of the OsMYBR1 was cloned into the pGreenⅡ 62-SK vector as effector. Protoplast preparation and transformation were conducted according to Zhong et al. [34]. Primer details are in Supplemental Table S2. The CDS of OsMYBR1 was amplified from cDNA of NIP and inserted into the pGEX6P-1 vector to obtain the GST-OsMYBR1 fusion protein. EMSA were performed using a EMSA Kit according to manufacturer instruction (GS009; Beyotime, Shanghai, China).

### 2.9. Statistical Analysis

Statistical significance of inter-group differences was assessed using one-way ANOVA and post hoc Tukey’s multiple comparisons test, conducted with Prism software version 9.0 (GraphPad, San Diego, CA, USA). Significant differences are indicated by different letters at *p* < 0.05. Statistical significance of two groups was assessed using Student’s *t*-test in R software version 4.0.5 with the “pwr” package (* *p* < 0.05, ** *p* < 0.01).

## 3. Results

### 3.1. Identification and Characterization of MYB Proteins in Rice

In this study, we identified 137 MYB family proteins containing MYB domain in rice by utilizing HMMER analysis to search the *Oryza sativa* L. ssp. *Japonica* protein database (E-value < 1.2 × 10^−28^) (Supplementary Table S1). Based on the number of MYB binding domains, the MYB genes can be classified into four subgroups: MYB-related genes (1R-MYB), R2R3-MYB (2R-MYB), R1R2R3-MYB (3R-MYB), and atypical MYB genes (4R-MYB) (Supplementary Table S1). Notably, the R2R3-MYB subfamily has the largest number of genes, accounting for 78.1% of the total MYB genes in rice (Supplementary Table S1). We performed an expression profile analysis of these 137 MYB transcription factors using RNA-seq data from the Rice Expression Database (https://ngdc.cncb.ac.cn/red/ (accessed on 20 February 2025)). The results showed that nine MYB genes (*LOC_Os05g37060* (1R-MYB), *LOC_Os05g28320* (R2R3-MYB), *LOC_Os07g31470* (R2R3-MYB), *LOC_Os01g74590* (R2R3-MYB), *LOC_Os06g14710* (1R-MYB), *LOC_Os03g25304* (1R-MYB), *LOC_Os10g20990* (1R-MYB), *LOC_Os02g42850* (R2R3-MYB), *LOC_Os04g45020* (R2R3-MYB)) were highly or specifically expressed in immature rice seeds (Figure S1).

### 3.2. Gene Expression Patterns, Subcellular Localization, and Phylogenetic Analysis of the OsMYBR1 Protein

**Figure 1:**
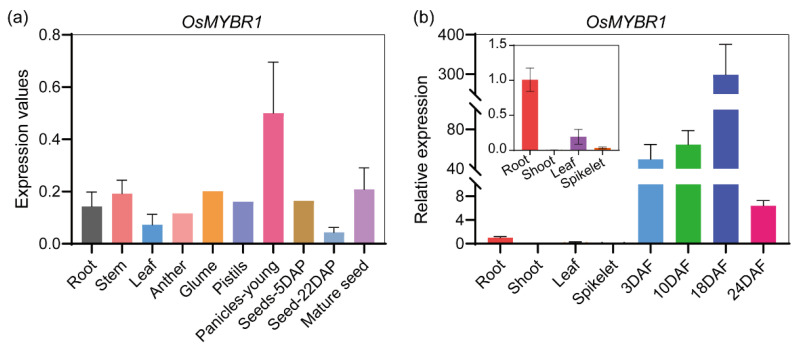
The expression pattern of *OsMYBR1* in rice. (**a**) The transcripts per million (TPM) value of *OsMYBR1* in different tissues of rice. The expression values of *OsMYBR1* obtained from RiceData (www.ricedata.cn), in which each gene model was quantified as TPM using Kallisto (v0.51.0). DAP is days after pollination of seeds. (**b**) qRT-PCR analysis of *OsMYBR1* in different tissues and in developing seeds of wild-type NIP. DAF is days after flowering of seeds. The 3DAF, 10DAF, and 18DAF samples collectively represent immature seeds. For rice seeds, DAF is equivalent to DAP.

**Figure 2:**
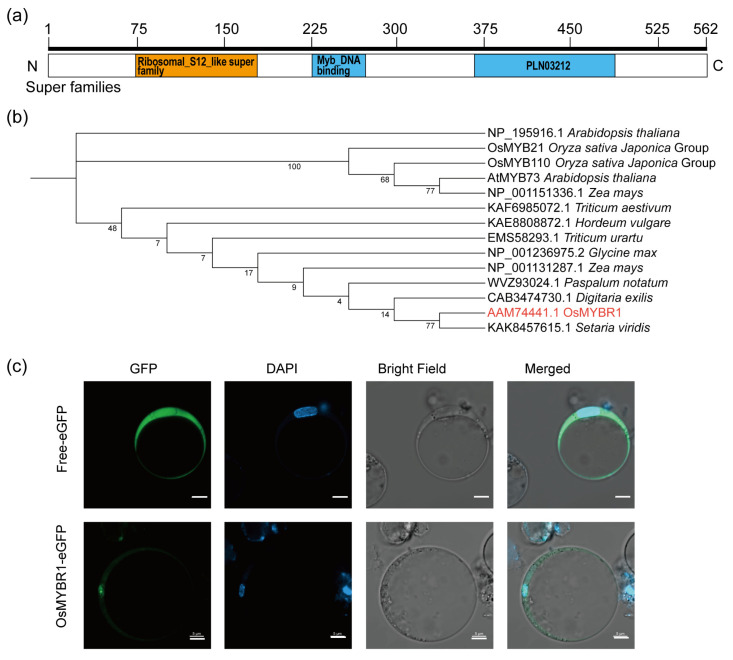
Prediction of conserved domains in OsMYBR1, phylogenetic analysis of OsMYBR1, and analysis of the subcellular location of OsMYBR1. (**a**) Search for conserved domains of OsMYBR1 in NCBI (https://www.ncbi.nlm.nih.gov/Structure/cdd/wrpsb.cgi (accessed on 5 March 2025)). (**b**) Phylogenetic tree of OsMYBR1 (in red) and its orthologues. The phylogenetic tree was constructed using the neighbor-joining algorithm and bootstrap method with 1000 replicates. (**c**) Subcellular localization of OsMYBR1-eGFP in rice protoplasts (bottom panel). Bars = 5 μm.

Among the nine MYB genes above, we selected the *LOC_Os10g 20990*, named as *OsMYBR1*, to further study. The qRT-PCR analysis indicated that *OsMYBR1* was mainly expressed in immature rice seed, which was consistent with the prediction of the Rice Expression Database (Figure 1). Protein sequence analysis by using NCBI-CDD (https://www.ncbi.nlm.nih.gov/cdd (accessed on 5 March 2025)) revealed that OsMYBR1 contains a ribosomal S12-like superfamily domain, a Myb-like DNA-binding domain, and a PLN03212 superfamily domain (Figure 2a). Phylogenetic analysis showed that KAK8457615.1, a ribosomal S12-like superfamily protein in *Setaria viridis*, shares the highest homology with OsMYBR1 (Figure 2b). However, protein sequence alignment indicates that OsMYBR1 shares only 16% similarity with KAK8457615.1, primarily within the ribosomal S12-like superfamily domain (Figure S2). The closest orthologues in *Arabidopsis* are NP_195916.1 and AtMYB73; NP_195916.1 shares similarity mainly in the ribosomal S12-like superfamily domain, while AtMYB73 shares similarity primarily in the Myb-like DNA-binding domain (Figures S2 and S3). These results suggest that OsMYBR1 is a specific protein characterized by a ribosomal S12-like superfamily, a Myb-like DNA-binding domain, and a PLN03212 superfamily domain.

Subcellular localization analysis revealed that OsMYBR1-eGFP fluorescence signals were detected in both the cytoplasm and nucleus of rice protoplasts, similar to the free eGFP control (Figure 2c), indicating that OsMYBR1 localizes to both compartments.

### 3.3. Loss-of-Function of OsMYBR1 Alters Starch Biosynthesis in Rice Endosperm

**Figure 3:**
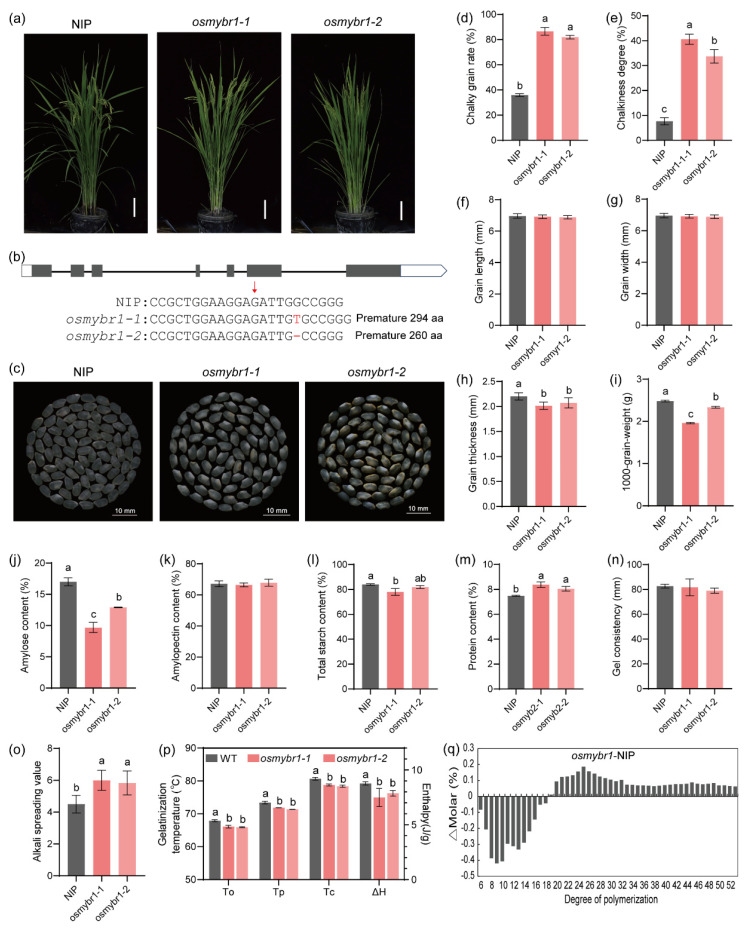
Phenotypical characterization of *osmybr1* and wild type NIP (**a**). Plant morphology at grain-filling stage (**b**). Generation of *osmybr1* mutants via CRISPR/Cas9-mediated genome editing. The red arrow indicates the target region of OsMYBR1, with the mutated sites highlighted in red. (**c**). Grains appearance of *osmybr1* and NIP (**d**–**i**). Comparison of the chalky grain rate (**d**), chalkiness degree (**e**), seed length (**f**), width (**g**), thickness (**h**), and 100-grain-weight (**i**) of *osmybr1* and NIP. (**j**–**l**), amylose content (**j**), amylopectin content (**k**), total starch content (**l**), and protein content (**m**) in mature seeds of *osmybr1* and NIP (**n**). Gel consistency of starches from NIP and the *osmybr1* mutants (**o**). Alkali spreading vale in endosperms of NIP and the *osmybr1* mutants (**p**). Differential scanning calorimetry (DSC) analysis: onset temperature (To), peak temperature (Tp), conclusion temperature (Tc), and gelatinization enthalpy (ΔH) (**q**). Amylopectin chain length distribution differences between NIP and *osmybr1*. Data presented means ± SD of three replicates. Different letters denote significant differences (*p* < 0.05, one-way ANOVA with two-sided Tukey’s HSD test).

To investigate OsMYBR1 function, *osmybr1* mutants were generated via a CRISP/Cas9 system in the *japonica* cultivar ‘Nipponbare’. Two homozygous mutants were obtained in T1 generation (Figure 3a,b). DNA sequencing analysis revealed that *osmybr1-1* contained a 1 bp insertion, leading to a premature stop codon and a truncated 294-amino acid protein. *osmybr1-2* has a 1 bp deletion producing a 260-amino acid truncated protein (Figure S4). Compared with NIP, the chalky grain rate and chalkiness degree of the *osmybr1-1* and *osmybr1-2* were significantly increased (Figure 3c–e). SEM analysis of mature endosperm cross-sections showed loosely packed, spherical starch granules with large air spaces in mutants, unlike the densely packed, irregular polyhedral granules in NIP (Figure S5). Grain length and width did not differ significantly, but mutant grains were thinner, leading to reduced 1000-grain weight (Figure 3f–i). The results suggest OsMYBR1 influences rice grain appearance and yield.

The amylose content in the *osmybr1* mutants was significantly lower compared to the wild type (Figure 3j), while the amylopectin content showed no significant difference (Figure 3k). The total starch content also slightly decreased in the mutants (Figure 3l). Notably, the protein content shows a slight increase in the mutants compared to wild type (Figure 3m). The starch gel consistency did not differ significantly between NIP and *osmybr1* mutants (Figure 3n). However, the alkali spreading value (ASV) significantly increased in mutants (Figure 3o), indicating that the loss of OsMYBR1 function made rice more easily gelatinized.

We further analyzed the thermal properties of the two *osmybr1* mutants and NIP using differential scanning calorimetry (DSC). Onset temperature indicates the start of amylopectin crystallite melting, while gelatinization enthalpy reflects the heat required for this process [19]. The mutants exhibited significantly lower I onset, peak, and end gelatinization temperatures, as well as decreased gelatinization enthalpy (Figure 3p), suggesting that OsMYBR1 influences rice gelatinization characteristics.

Amylopectin includes chains of the types A, B1, B2, and B3, with size ranges of DP (degree of polymerization) ~6–12, DP = 13 to 24, DP = 25 to 36, and DP ≥ 37, respectively [35]. To assess the impact of *osmybr1* mutants on amylopectin structure, we analyzed chain length distribution via high-performance anion-exchange chromatography (HPAEC). The mutants showed a significant reduction in short chains (DP 6–18) and an increase in longer chains (DP 19–52) compared to NIP (Figure 3q). The calculation of the ratios for each type in the amylopectin of NIPand *osmybr1* mutants showed that short chains (type A) and short B1 chains were much less prevalent in *osmybr1* plants compared to the wild type, while longer B1 chains and long chain B2–3 were more abundant in the *osmybr1* plants. The results suggested that the mutation of OsMYBR1 did not affect the content of amylopectin, but influenced its chain length distribution.

### 3.4. Mutation of OsMYBR1 Affects the Expression of SSEGs

**Figure 4:**
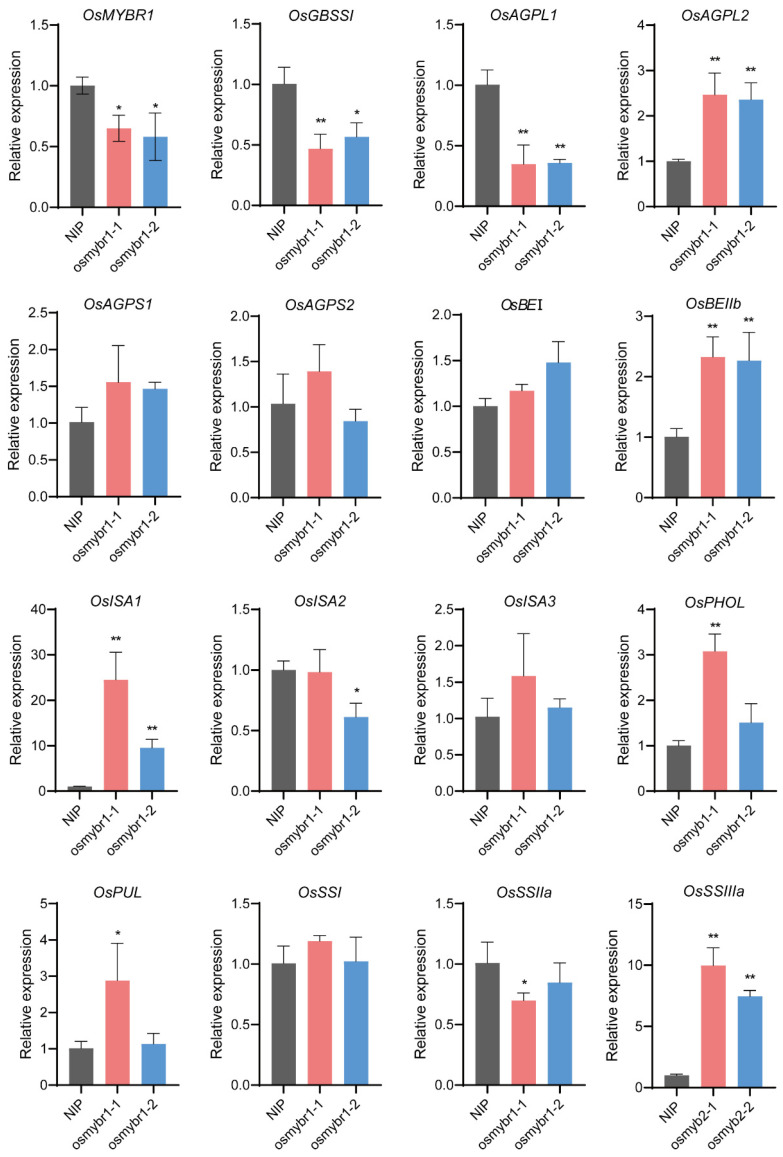
Expression of starch synthesis enzyme-coding genes in NIP and the *osmybr1* mutants analyzed by qRT-PCR. Data represent mean ± SD of three biological replicates. * *p* < 0.05, ** *p* < 0.01, Student’s *t*-test.

We analyzed *OsMYBR1* expression in wild type NIP and *osmybr1* mutants, finding significantly lower transcript levels in mutants (Figure 4). Since starch is the main component of rice endosperm and the roles of SSEGs in the process of starch synthesis are well understood, we investigated their expression in both *osmybr1* mutants and wild type NIP. Among the 15 endosperm-preferentially expressed SSEGs [22], except for *OsAGPS1*, *OsAGPS2*, *OsISA2*, *OsISA3*, *OsBEⅠ*, *OsPUL*, *OsSSⅠ*, and *OsSSⅡa* showed no significant differences in expression levels; the expression levels of genes *OsGBSSⅠ* and *OsAGPL1* was significantly down-regulated, while the expression of *OsAGPL2*, *OsBEⅡb*, *OsISA1*, *OsPHOL*, and *OsSSⅢa* were up-regulated in *osmybr1* (Figure 4). These results indicated that OsMYBR1 could regulate the development of endosperm by modulating the expression of several genes related to starch synthesis.

### 3.5. OsMYBR1 Regulates Six SSEGs by Directly Binding to Their Promoters

**Figure 5:**
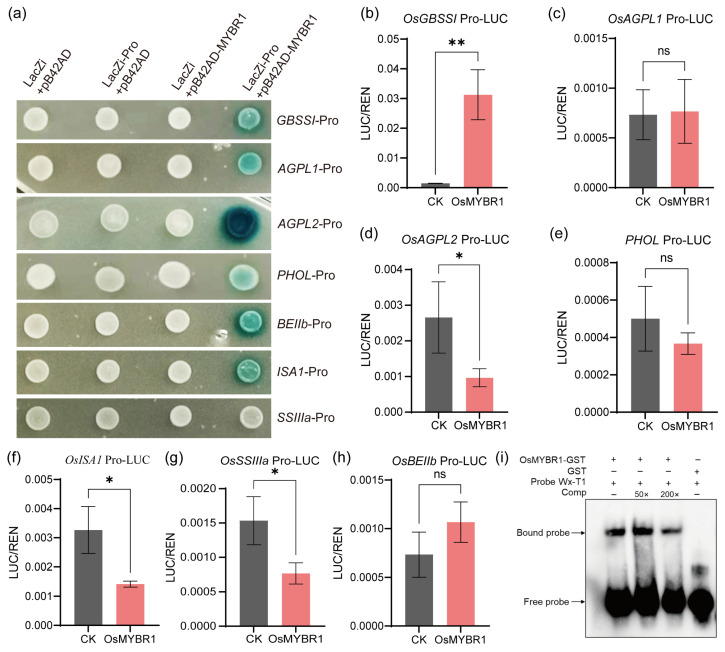
OsMYBR1 bind and regulate several SSEG expressions. (**a**) Yeast one-hybrid assay showing interaction between OsMYBR1 and the promoters of seven SSEGs. (**b**–**h**) Transcription assay of OsMYBR1 for *OsGBSSI*, *OsAGPL1*, *OsAGPL2*, *PHOL*, *OsISA1*, *OsSSⅢa*, and *OsBEⅡb* in rice protoplasts. The LUC reporter genes were driven by ~2 kb upstream promoter regions. Relative luciferase activities (LUC/REN) were measured following co-transfection with effector and reporter plasmids; empty 62SK vector served as negative control. Data represent mean ± SD of three biological replicates. Statistical significance was evaluated using Student’s *t*-test: *p* < 0.05 (*), *p* < 0.01 (**), and ns indicates no significant difference. (**i**) EMSAs showing OsMYBR1 binding to the *OsGBSSI* promoter. Black arrowheads indicate the shifted bands (up) and the free probe bands (down). The probe sequence was ‘AGTTGGCAGGCACTAATAGCTACAGTAAAGTAAAGAGCAACGTGCC’.

The qRT-PCR results indicate that several SSEGs (*OsGBSSⅠ*, *OsAGPL1*, *OsAGPL2*, *OsPHOL*, *OsBEⅡb*, *OsISA1*, *and OsSSⅢa*) are regulated by *OsMYBR1*. We first conducted Y1H and LUC reporter assays to validate this regulation. The Y1H analysis revealed that, except *OsSSⅢa*, OsMYBR1 could directly bind to the promoters of the other six SSEGs (*OsGBSSⅠ*, *OsAGPL1*, *OsAGPL2*, *OsPHOL*, *OsBEⅡb*, and *OsISA1*) (Figure 5a). The LUC results indicated that OsMYBR1 enhances the expression of *OsGBSSⅠ* while repressing the expression of *OsAGPL2*, *OsISA1*, and *OsSSⅢa*; the other three SSEGs showed no significant change in expression (Figure 5b–h). Additionally, an EMSA analysis confirmed that OsMYBR1 directly binds to the promoter of *OsGBSSⅠ* (Figure 5i). These results suggest that OsMYBR1 could directly bind to the promoters of six SSEGs (*OsGBSSⅠ*, *OsAGPL1*, *OsAGPL2*, *OsPHOL*, *OsBEⅡb*, and *OsISA1*), enhancing the expression of *OsGBSSⅠ* while repressing the expression of *OsAGPL2*, *OsISA1*, and *OsSSⅢa*.

### 3.6. Overexpression of OsMYBR1 Enhances Rice ECQ Without Affecting Endosperm Appearance

**Figure 6:**
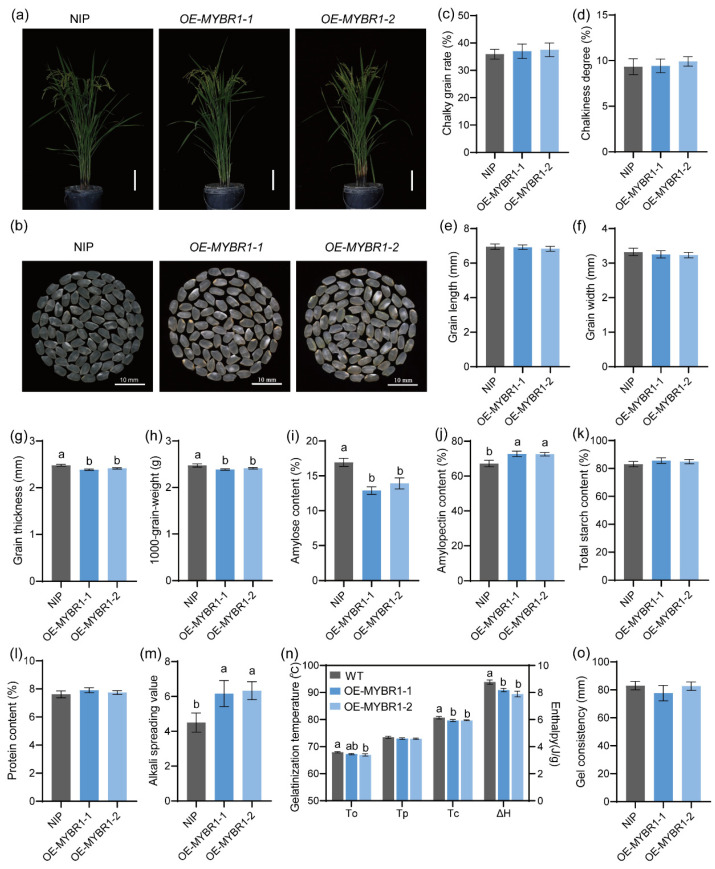
Phenotypical characterization of wild type NIP and the over-expression *OsMYBR1* lines. (**a**) Plant morphology at grain-filling stage. (**b**–**d**): Comparison of grains appearance (**b**), chalky grain rate (**c**), and chalkiness degree (**d**) of NIP and the *OsMYBR1* over-expression lines. (**e**–**h**): omparison of the seed length (**e**), width (**f**), thickness (**g**), and 100-grain-weight (**h**) of NIP and the *OsMYBR1* over-expression lines. (**i**–**l**): Amylose content (**i**), amylopectin content (**j**), total starch content (**k**), and protein content (**l**) in mature seeds of NIP and the *OsMYBR1* over-expression lines. (**m**) Alkali spreading value in endosperms of NIP and the *OsMYBR1* over-expression lines. (**n**) DSC analysis including onset temperature (To), peak temperature (Tp), conclusion temperature (Tc), and enthalpy of gelatinization (ΔH). (**o**) Gel consistency of endosperm starch in OsMYBR1 overexpression lines and wild type. All data represent mean ± SD of three replicates (**a**,**c**,**d**,**f**–**n**). Different letters denote significant differences (*p* < 0.05, one-way ANOVA with two-sided Tukey’s HSD test).

To evaluate the application value of OsMYBR1, we generated overexpression transgenic lines. Three overexpression lines, OE-7, OE-15, and OE-16, were identified via qRT-PCR (Figure S6). The T2 generations of OE-15 and OE-16, named as OE-MYBR1-1 and OE-MYBR1-2, were planted in the experiment field for phenotype analysis. Compared to NIP, OE lines showed no significant differences in major agronomic traits, grain appearance (chalky grain rate and chalkiness degree), or grain phenotype (grain length and grain width) compared to the NIP (Figure 6a–f). However, grain thickness slightly decreased in the *OsMYBR1*-overexpression lines, leading to reduced grain weight compared to NIP (Figure 6g,h).

In terms of starch composition, amylose content significantly decreased in the *OsMYBR1*-overexpression lines compared to NIP (Figure 6i), while amylopectin content significantly increased (Figure 6j). Total starch content in the *OsMYBR1*-overexpression lines showed a slight increase but was not significant from NIP (Figure 6k). The protein content also showed no significant difference between *OsMYBR1*-overexpression lines and NIP (Figure 6l). The ASV significantly increased in *OsMYBR1*-overexpression lines, indicating that these lines gelatinized more easily (Figure 6m). DSC analysis revealed significantly lower conclusion temperature and gelatinization enthalpy, with slight, non-significant decreases in onset and peak temperatures in *OsMYBR1*-overexpression lines (Figure 6n). These results further demonstrated that the *OsMYBR1*-overexpression lines gelatinized more easily than NIP. The gel consistency showed no significant difference when compared to NIP (Figure 6o). In summary, the overexpression of *OsMYBR1* decreased amylose content and gelatinization temperature while increased amylopectin content, without affecting grain appearance. These findings suggest that *OsMYBR1* overexpression could be utilized to improve rice quality.

## 4. Discussion

MYB family transcription factors regulate diverse processes including secondary metabolism [26], hormone signaling [25,36], and stress response [27]. In maize, MYBs such as ZmMYB14 activate starch synthesis genes including *ZmBT1* [29], and ZmMYB138/115 are candidates linked to starch biosynthesis [28]. However, there are few reports on MYB family genes involved in the starch synthesis of rice. This study identified the 1R-MYB transcription factor *OsMYBR1* (LOC_Os10g20990) as a regulator of starch synthesis in rice endosperm. OsMYBR1 is a specifical MYB-related protein that contains a ribosomal S12-like superfamily domain, a Myb-like DNA-binding domain, and a PLN03212 superfamily domain, localizing in both the cell nucleus and cytoplasm.

*OsMYBR1* regulates amylose content in rice grains. The *osmybr1* mutants exhibited significantly lower amylose content (9.7% and 12.9%) compared to wild type NIP (17.0%). The *Waxy* (*Wx*) gene, encoding granule-bound starch synthase Ⅰ (OsGBSSⅠ) controls amylose synthesis [6,7,8,9]. Our Y1H, LUC, and EMSA assays demonstrated that OsMYBR1 directly binds to the *OsGBSSⅠ* promoter and enhances its expression (Figure 5). This was further confirmed by qRT-PCR, indicating that *OsMYBR1* mediates amylose biosynthesis in rice by regulating *OsGBSSⅠ* expression. Loss-of-function of *OsMYBR1* reduce expression of *OsAGPL1*, a subunit of ADP-glucose pyrophosphorylase (AGPase), which is a heterotetramer composed of two large subunits (AGPL) and two small subunits (AGPS) catalyzing the first committed step in starch biosynthesis [3,37,38]. Although *OsAGPL2* expression increased, the reduced *OsAGPL1* likely impaired the formation of AGPase, leading to decreased ADP-glucose availability for starch synthesis in *osmybr1* mutants. Consequently, both starch content and grain yield were reduced.

*OsMYBR1* affects the amylopectin chain length distribution. Amylopectin comprises chains classified as types A, B1, B2, and B3, ranging in size from DP ~6–12, DP = 13 to 24, DP = 25 to 36, and DP ≥ 37, respectively [35]. Amylopectin synthesis involves SSs, SBEs, and DBEs, which elongate amylopectin chains, generate branches, and remove improper branches [37]. The role of PHOL remains unclear, with debate over whether it plays a synthetic versus phosphorolytic role in vivo [39]. *phol* mutants show a higher proportion of short chains (DP ≤ 11) and a lower proportion of intermediate chains (DP 13–21) [39]. BEIIb specifically synthesizes short chains; *beIIb* mutants increase long branch-chains and reduce short branch-chains [14,15]. OsISA1 is responsible for removing excessive or misplaced branch points in amylopectin. *osisa1* mutants show increased short A chains (DP ≤ 12) and intermediate B2 chains (25 ≤ DP ≤ 36), alongside a reduction in B1 (13 ≤ DP ≤ 24) and longer B3 to B4 chains (DP ≥ 37) compared to wild type [16]. Chao et al. [17] found that in *isa1* mutants, short chains (DP ≤ 10) increased while longer chains (DP 10–60) decreased. OsSSIIIa is crucial for generating relatively long chains in rice endosperm, especially those with DP ≥ 30 and DP 16 to 20 [12,13]. In *osmybr1* mutants, expression of *OsPHOL*, *OsBEⅡb*, *OsISA1*, and *OsSSⅢa* were significantly upregulated, with increases of over 9.5- and 7.5-fold for *OsISA1* and *OsSSⅢa*, respectively. Elevated *OsSSⅢa* expression may boost the content of long chains (DP ≥ 30), and upregulation of *OsISA1* expression can remove excessive or misplaced branch points, increasing the longest B3 to B4 chains (DP ≥ 37). These changes led to higher proportions of longer B1 and B2–3 chains (DP 19–52), and decreased proportions of A chains and short B1 chains (DP 6–18) in *osmybr1* mutants (Figure 3q). The changes in amylopectin chain length distribution in *osmybr1* mutant (in which *OsISA1* and *OsSSⅢa* were upregulated) were similar to those of *OsISA1* and *OsSSⅢa*, indicating the combined effects of multiple starch synthesis genes.

Mutations in *OsMYBR1* altered the physicochemical properties of endosperm starch. The *Wx* gene is a major regulator of amylose content and gel consistency regulation and also contributes to rice gelatinization temperature [40]. Changes in amylopectin structure can affect starch granules crystallinity, altering the gelatinization property. A chains are critical for crystalline structure formation; alterations in short A chains affect gelatinization [22]. Mutation of *BEIIb* increases the proportion of long branch-chains, reduces short branch-chains, and raises gelatinization temperature [41]. Conversely, *sbe1* mutants exhibit increased short chains, decreased long chains, and lower gelatinization temperature [42]. In *osmybr1* mutants, reduced A chains coincided with lower gelatinization temperatures, differing from previous reports, but consistent with Fu et al. [22] for *rsr1* mutants, which showed decreased A chains and increased B1 and B2–3 chains.

**Figure 7:**
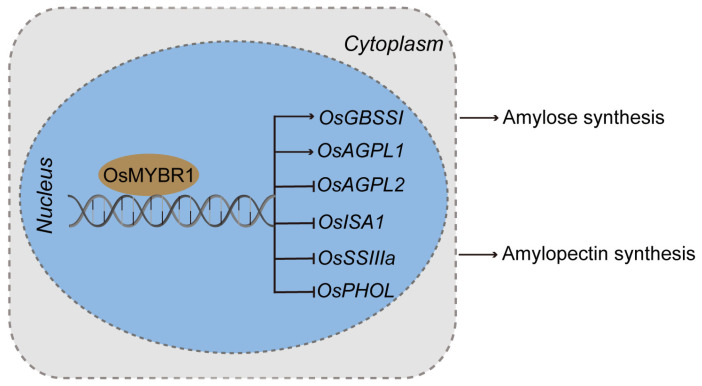
A model for the OsMYBR1 regulates the rice starch synthesis.

Amylose content, gel consistency, gelatinization temperature, and viscosity are widely accepted indicators for assessing rice ECQ [40,43]. Our results indicate that *osmybr1* mutations decrease amylose content and gelatinization temperature, along with a lower enthalpy (ΔH) value, making starch easier to gelatinize, indicated that the mutation of *OsMYBR1* improves rice ECQ. While gel consistency remained unchanged, increased chalkiness and chalky grain rate adversely affected rice appearance, impacting overall rice quality. Overexpression of OsMYBR1 improved rice ECQ without affecting endosperm appearance. We found that overexpression-OsMYBR1 increased the amylopectin and total starch content while decreasing amylose content. This indicates that both OsMYBR1 mutation and overexpression lead to reduced amylose content. This seemingly paradoxical phenomenon has also been reported in other studies. For example, OsSGL (STRESS_tolerance and GRAIN_LENGTH), a DUF1645 domain protein, regulates several starch biosynthesis genes including *OsISA1*, *PHOL*, *OsAGPL1*, *OsSBE1*, *OsBEⅡb*, *OsSUS1*, and *OsSSⅡa*, thereby balancing starch content in rice seeds. Both the overexpression and knockdown of *OsSGL* reduced the amylose and starch content in the rice grains [44]. Similarly, SDG711, a histone H3K27me2/3 transmethylase in rice, directly binds to the gene bodies of several starch synthesis and amylase genes. Both the overexpression and downregulation of *SDG711* result in decreased amylose and starch content in rice grains [45]. Based on these examples, OsMYBR1 likely functions as a regulator of starch metabolism, maintaining the balance of starch content in rice seeds. OsMYBR1 regulates the expression of seven SSEGs (*OsGBSSⅠ*, *OsAGPL1*, *OsAGPL2*, *OsPHOL*, *OsBEⅡb*, *OsISA1*, and *OsSSⅢa*) (Figure 7), but the mechanism underlying the decrease in amylose content in overexpression lines requires further investigation. Overexpression of OsMYBR1 resulted in an increased alkali spreading value (ASV) and decreased the enthalpy values (ΔH), indicating that the endosperm starch more easily gelatinized, even though gelatinization temperature and 1000-grain weight showed slight decreases. Importantly, starch granule morphology and endosperm appearance remained unchanged in *OsMYBR1* overexpression lines. Overall, overexpression of *OsMYBR1* can be utilized to improve rice quality by fine-tuning starch synthesis in rice grains. But, when considering biosafety, genome editing offers a safer approach than transgenic overexpression for genetic improvement. Future work will focus on haplotype analysis of *OsMYBR1* in rice germplasms and association studies with phenotypes to identify beneficial haplotypes for use in rice breeding.

## 5. Conclusions

This study identified an 1R-MYB family gene, *OsMYBR1*, that regulates the expression of seven SSEGs (*OsGBSSⅠ*, *OsAGPL1*, *OsAGPL2*, *OsPHOL*, *OsBEⅡb*, *OsISA1*, and *OsSSⅢa*), thereby modulating starch biosynthesis in rice endosperm. Overexpression of OsMYBR1 increased amylopectin and total starch content while decreasing amylose content, resulting in a higher alkali spreading value (ASV) and lower enthalpy value (ΔH), indicating that the endosperm starch more easily gelatinized. Despite changes in starch composition and physicochemical properties, starch granule morphology and endosperm appearance were unaffected in *OsMYBR1* overexpression lines. Overall, overexpression of *OsMYBR1* can be utilized to improve rice ECQ by fine-tuning starch synthesis in rice grains.

## Data Availability

Data is contained within the article and Supplementary Materials.

## Supplementary Materials

The following supporting information can be downloaded at: https://www.mdpi.com/article/10.3390/life15060962/s1, Figure S1: The expression pattern of MYB family genes; Figure S2. Amino acid sequence alignment of OsMYBR1 with four homologous proteins; Figure S3. Amino acid sequence alignment of OsMYBR1 and three homologous proteins; Figure S4. Amino acid sequence alignment of OsMYBR1, osmybr1-1 and osmybr1-2. The osmybr1-1 mutant contained a 1 bp insertion that introduces a premature stop codon, resulting in a truncated 294-amino acid protein. The osmybr1-2 mutant has a 1 bp deletion, producing a truncated protein of 260-amino acids. Both mutations disrupt the Myb-like binding domain and leads to the loss of the PLN03212 superfamily domain; Figure S5. Scanning electron microscopy images of mature grains in NIP, osmybr1 and overexpression *OsMYBR1*. Scale bars, 10 μm; Figure S6. qRT-PCR analysis of *OsMYBR1* overexpression lines; Table S1: A total of 137 MYB family proteins were identified in rice; Table S2: Primers used in this study.
