# Supplementary material for: OsMYBR1, a 1R-MYB Family Transcription Factor Regulates Starch Biosynthesis in Rice Endosperm
Source: Life (Basel). 2025 Jun 16;15(6):962. doi: 10.3390/life15060962 (PMC12194225; doi:10.3390/life15060962)
Supplement: Supplementary file 1 [file life-15-00962-s001.zip › Supplement Figures.pdf]

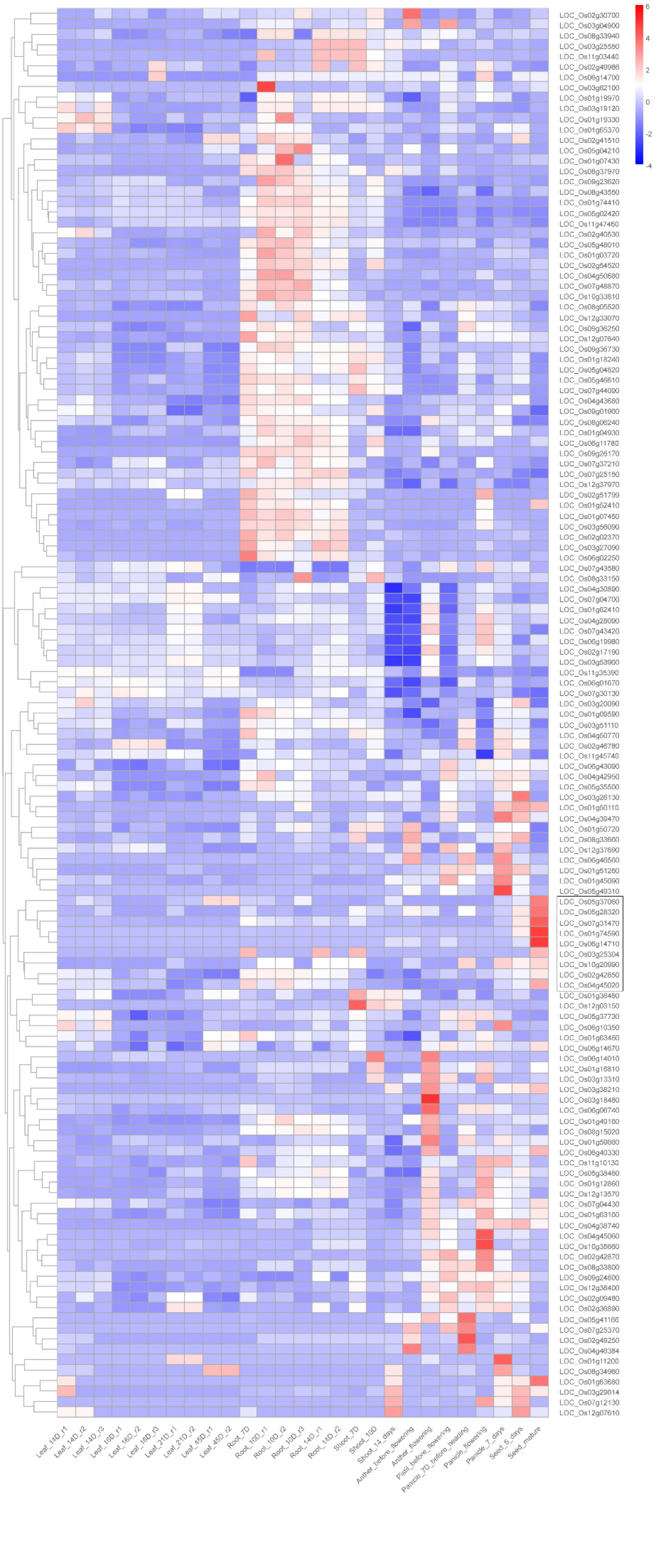
6
4
2
0
-2
-4
LOC_Os02g30700
LOC_Os03g04900
LOC_Os08g33940
LOC_Os03g25550
LOC_Os11g03440
LOC_Os02g49986
LOC_Os06g14700
LOC_Os03g62100
LOC_Os01g19970
LOC_Os03g19120
LOC_Os01g19330
LOC_Os01g65370
LOC_Os02g41510
LOC_Os05g04210
LOC_Os01g07430
LOC_Os08g37970
LOC_Os09g23620
LOC_Os08g43550
LOC_Os01g74410
LOC_Os05g02420
LOC_Os11g47460
LOC_Os02g40530
LOC_Os05g48010
LOC_Os01g03720
LOC_Os02g54520
LOC_Os04g50680
LOC_Os07g48870
LOC_Os10g33810
LOC_Os08g05520
LOC_Os12g33070
LOC_Os09g36250
LOC_Os12g07640
LOC_Os09g36730
LOC_Os01g18240
LOC_Os05g04820
LOC_Os05g46610
LOC_Os07g44090
LOC_Os04g43680
LOC_Os09g01960
LOC_Os08g06240
LOC_Os01g04930
LOC_Os06g11780
LOC_Os09g26170
LOC_Os07g37210
LOC_Os07g25150
LOC_Os12g37970
LOC_Os02g51799
LOC_Os01g52410
LOC_Os01g07450
LOC_Os03g56090
LOC_Os02g02370
LOC_Os03g27090
LOC_Os06g02250
LOC_Os07g43580
LOC_Os08g33150
LOC_Os04g30890
LOC_Os07g04700
LOC_Os01g62410
LOC_Os04g28090
LOC_Os07g43420
LOC_Os06g19980
LOC_Os02g17190
LOC_Os03g53960
LOC_Os11g35390
LOC_Os06g01670
LOC_Os07g30130
LOC_Os03g20090
LOC_Os01g09590
LOC_Os03g51110
LOC_Os04g50770
LOC_Os02g46780
LOC_Os11g45740
LOC_Os06g43090
LOC_Os04g42950
LOC_Os05g35500
LOC_Os03g26130
LOC_Os01g50110
LOC_Os04g39470
LOC_Os01g50720
LOC_Os08g33660
LOC_Os12g37690
LOC_Os06g46560
LOC_Os01g51260
LOC_Os01g45090
LOC_Os05g49310
LOC_Os05g37060
LOC_Os05g28320
LOC_Os07g31470
LOC_Os01g74590
LOC_Os06g14710
LOC_Os03g25304
LOC_Os10g20990
LOC_Os02g42850
LOC_Os04g45020
LOC_Os01g36460
LOC_Os12g03150
LOC_Os05g37730
LOC_Os06g10350
LOC_Os01g63460
LOC_Os06g14670
LOC_Os06g14010
LOC_Os01g16810
LOC_Os03g13310
LOC_Os03g38210
LOC_Os03g18480
LOC_Os06g06740
LOC_Os01g49160
LOC_Os08g15020
LOC_Os01g59660
LOC_Os06g40330
LOC_Os11g10130
LOC_Os05g38460
LOC_Os01g12860
LOC_Os12g13570
LOC_Os07g04430
LOC_Os01g63160
LOC_Os04g38740
LOC_Os04g45060
LOC_Os10g35660
LOC_Os02g42870
LOC_Os08g33800
LOC_Os09g24800
LOC_Os12g38400
LOC_Os02g09480
LOC_Os02g36890
LOC_Os05g41166
LOC_Os07g25370
LOC_Os02g49250
LOC_Os04g46384
LOC_Os01g11200
LOC_Os08g34960
LOC_Os01g63680
LOC_Os03g29614
LOC_Os07g12130
LOC_Os12g07610
Leaf_14D_r1
Leaf_14D_r2
Leaf_14D_r3
Leaf_16D_r1
Leaf_16D_r2
Leaf_16D_r3
Leaf_21D_r1
Leaf_21D_r2
Leaf_45D_r1
Leaf_45D_r2
Root_7D
Root_10D_r1
Root_10D_r2
Root_10D_r3
Root_14D_r1
Root_14D_r2
Shoot_7D
Shoot_10D
Shoot_14_days
Anther_before_flowering
Anther_flowering
Pistil_before_flowering
Panicle_7D_before_heading
Panicle_flowering
Panicle_7_days
Seed_5_days
Seed_mature

Figure S1. The expression pattern of MYB family genes.

Spatio-temporal expression profile of the MYB family genes based on transcriptome databases in the IC4R (Information Commons for Rice; [Home - IC4R Expression (https://ngdc.cncb.ac.cn/red/)](https://ngdc.cncb.ac.cn/red/)).

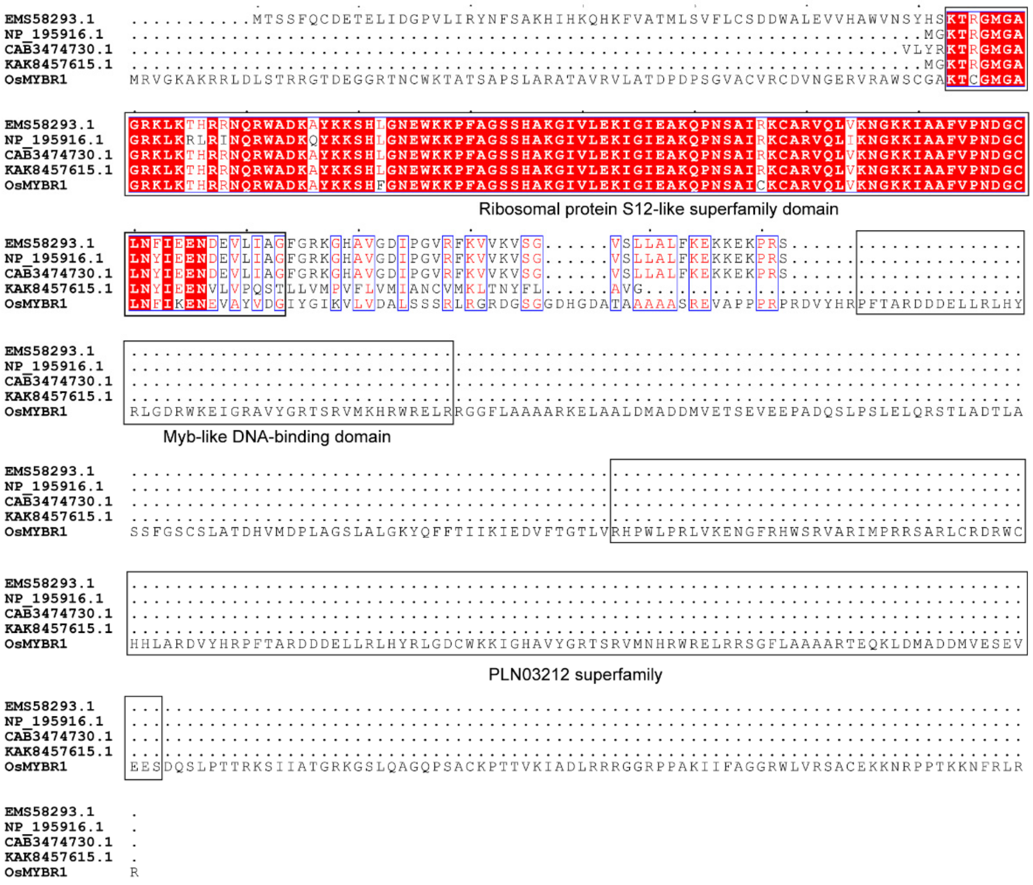

Figure S2. Amino acid sequence alignment of OsMYBR1 with four homologous proteins. KAK8457615.1, hypothetical protein SEVIR_3G218200v4 [*Setaria viridis*]; CAB3474730.1, unnamed protein product, partial [*Digitaria exilis*]; EMS58293.1, 40S ribosomal protein S23 [*Triticum urartu*]; NP_195916.1, Ribosomal protein S12/S23 family protein [*Arabidopsis thaliana*]. Protein sequences were aligned in the MEGA 7.0 software based on the MUSCLE method. The black box highlights domains related to the ribosomal protein S12-like family, Myb-like DNA-binding, and PLN03212 superfamily, respectively.

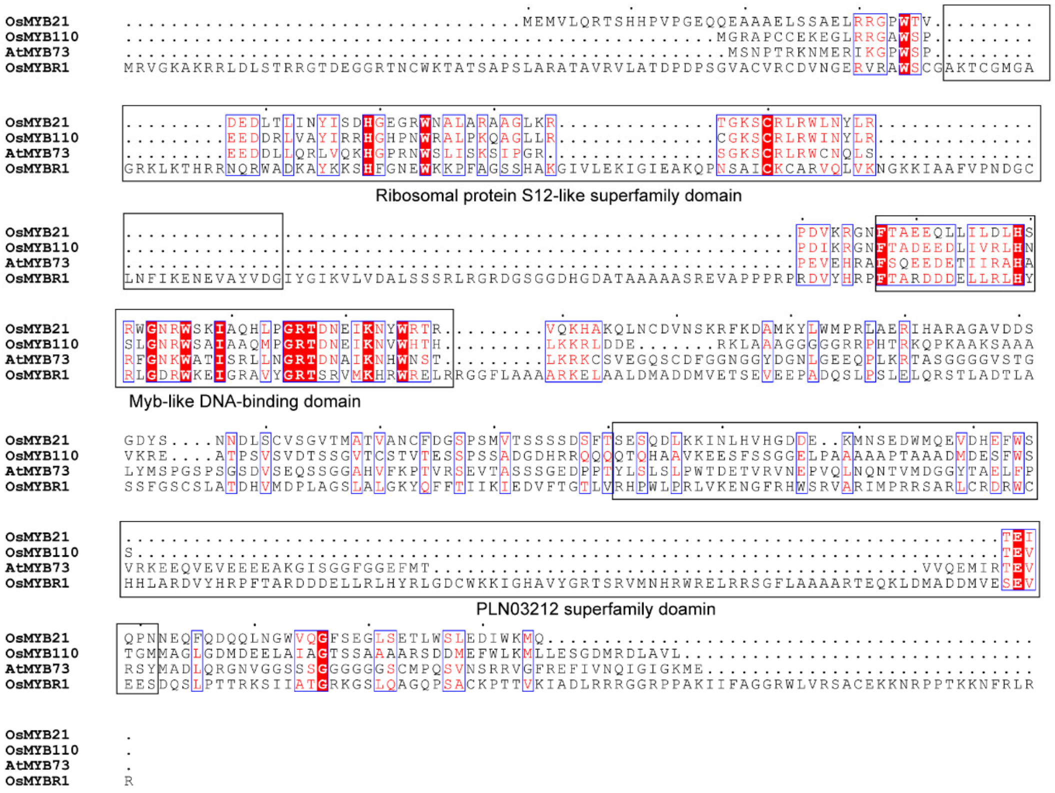

Figure S3. Amino acid sequence alignment of OsMYBR1 and three homologous proteins. AtMYB73, myb domain protein 73 [Arabidopsis thaliana]. Protein sequences were aligned in the MEGA 7.0 software based on the MUSCLE method. The black box highlights domains related to the ribosomal protein S12-like family, Myb-like DNA-binding, and PLN03212 superfamily, respectively.

```
                                                      250
OsMYBR1    MRVGKAKRRLDLSTRRGTDEGGRTNCWKTATSAPSLARATAVRVLATDPDPSGVACVRCDVNGERVRAW
osmybr1-1  MRVGKAKRRLDLSTRRGTDEGGRTNCWKTATSAPSLARATAVRVLATDPDPSGVACVRCDVNGERVRAW
osmybr1-2  MRVGKAKRRLDLSTRRGTDEGGRTNCWKTATSAPSLARATAVRVLATDPDPSGVACVRCDVNGERVRAW

OsMYBR1    SCGAKTCGMGAGRKLKTHRRNQRWADKAYKKSHFGNEWKKPFAGSSHAKGIVLEKIGIEAKQPNSAICK
osmybr1-1  SCGAKTCGMGAGRKLKTHRRNQRWADKAYKKSHFGNEWKKPFAGSSHAKGIVLEKIGIEAKQPNSAICK
osmybr1-2  SCGAKTCGMGAGRKLKTHRRNQRWADKAYKKSHFGNEWKKPFAGSSHAKGIVLEKIGIEAKQPNSAICK

OsMYBR1    CARVQLVKNGKKIAAFVPNDGCLNFIKENEVAYVDGIYGIKVLVDALSSSRLRGRDGSGGDHGDATAAA
osmybr1-1  CARVQLVKNGKKIAAFVPNDGCLNFIKENEVAYVDGIYGIKVLVDALSSSRLRGRDGSGGDHGDATAAA
osmybr1-2  CARVQLVKNGKKIAAFVPNDGCLNFIKENEVAYVDGIYGIKVLVDALSSSRLRGRDGSGGDHGDATAAA

                                                      250
OsMYBR1    AASREVAPPPRPRDVYHR[PFTARDDDELLRLHYRLGDRWKEIGRAVYGRTSRVMKHRWRELR]RGGFLAA
                                  Myb-line binding domain
osmybr1-1  AASREVAPPPRPRDVYHRPFTARDDDELLRLHYRLGDRWKEIVPGRLRPHIARHEAPLEGAPARRLPRC
osmybr1-2  AASREVAPPPRPRDVYHRPFTARDDDELLRLHYRLGDRWKEIAGPFTAAHRAS•••••••••••••••••

OsMYBR1    AARKELAALDMADDMVETSEVEEPADQSLPSLELQRSTLADTLASSFGSCSLATDHVMDPLAGSLALGK
osmybr1-1  CRKEGAGGARHGGRHGGN••••••••••••••••••••••••••••••••••••••••••••••••••••
osmybr1-2  ••••••••••••••••••••••••••••••••••••••••••••••••••••••••••••••••••••••

OsMYBR1    YQFFTIIKIEDVFTGTLVRHPWLPRLVKENGFRHWSRVARIMPRRSARLCRDRWCHHLARDVYHRPFTA
osmybr1-1  ••••••••••••••••••••••••••••••••••••••••••••••••••••••••••••••••••••••
osmybr1-2  ••••••••••••••••••••••••••••••••••••••••••••••••••••••••••••••••••••••

OsMYBR1    RDDDELLRLHYRLGDCWKKIGHAVYGRTSRVMNHRWRELRRSGFLAAAARTEQKLDMADDMVESEVEES
osmybr1-1  ••••••••••••••••••••••••••••••••••••••••••••••••••••••••••••••••••••••
osmybr1-2  ••••••••••••••••••••••••••••••••••••••••••••••••••••••••••••••••••••••

OsMYBR1    DQSLPTTRKSIIATGRKGSLQAGQPSACKPTTVKIADLRRRGGRPPAKIIFAGGRWLVRSACEKKNRPP
osmybr1-1  ••••••••••••••••••••••••••••••••••••••••••••••••••••••••••••••••••••••
osmybr1-2  ••••••••••••••••••••••••••••••••••••••••••••••••••••••••••••••••••••••

OsMYBR1    TKKNFRLRR
osmybr1-1  •••••••••
osmybr1-2  •••••••••
```

Figure S4. Amino acid sequence alignment of OsMYBR1, osmybr1-1 and osmybr1-2. The osmybr1-1 mutant contained a 1-bp insertion that introduces a premature stop codon, resulting in a truncated 294-amino acid protein. The osmybr1-2 mutant has a 1-bp deletion, producing a truncated protein of 260-amino acids. Both mutations disrupt the Myb-like binding domain and leads to the loss of the PLN03212 superfamily domain.

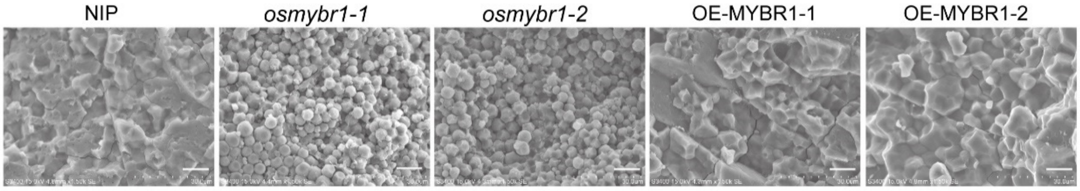

Figure S5. Scanning electron microscopy images of mature grains in NIP, *osmybr1* and overexpression OsMYBR1. Scale bars, 10 μm.

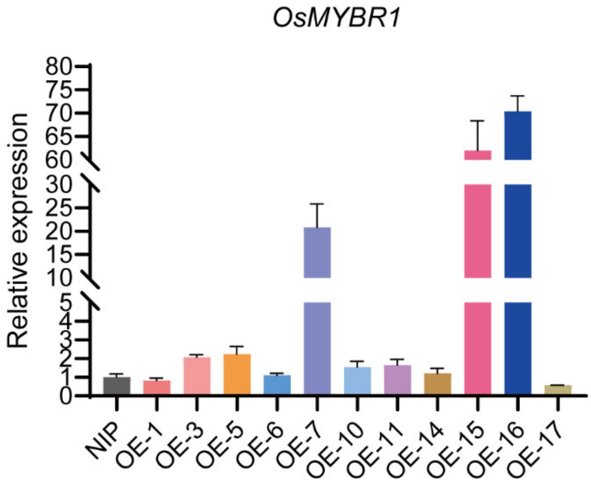

Figure S6. qRT-PCR analysis of *OsMYBR1* overexpression lines.

RNA extraction for qRT-PCR analysis was performed using leaves from the T0 generations of the *OsMYBR1* overexpression lines. The T2 generations of OE-15 and OE-16, designated as OE-MYBR1-1 and OE-MYBR1-2, were used for phenotypic characterization studies.
